# Molecular Characterization of a Novel Polymycovirus From *Penicillium janthinellum* With a Focus on Its Genome-Associated PASrp

**DOI:** 10.3389/fmicb.2020.592789

**Published:** 2020-10-20

**Authors:** Yukiyo Sato, Atif Jamal, Hideki Kondo, Nobuhiro Suzuki

**Affiliations:** ^1^Institute of Plant Science and Resources, Okayama University, Kurashiki, Japan; ^2^Crop Diseases Research Institute, National Agricultural Research Centre, Islamabad, Pakistan

**Keywords:** fungal virus, RNA virus, polymycovirus, *Penicillium janthinellum*, capsidless, multi-segmented, proline-alanine-serine rich protein

## Abstract

The genus *Polymycovirus* of the family *Polymycoviridae* accommodates fungal RNA viruses with different genomic segment numbers (four, five, or eight). It is suggested that four members form no true capsids and one forms filamentous virus particles enclosing double-stranded RNA (dsRNA). In both cases, viral dsRNA is associated with a viral protein termed “proline-alanine-serine-rich protein” (PASrp). These forms are assumed to be the infectious entity. However, the detailed molecular characteristics of PASrps remain unclear. Here, we identified a novel five-segmented polymycovirus, Penicillium janthinellum polymycovirus 1 (PjPmV1), and characterized its purified fraction form in detail. The PjPmV1 had five dsRNA segments associated with PASrp. Density gradient ultracentrifugation of the PASrp-associated PjPmV1 dsRNA revealed its uneven structure and a broad fractionation profile distinct from that of typical encapsidated viruses. Moreover, PjPmV1-PASrp interacted *in vitro* with various nucleic acids in a sequence-non-specific manner. These PjPmV1 features are discussed in view of the diversification of genomic segment numbers of the genus *Polymycovirus*.

## Introduction

Capsidless RNA viruses have been frequently found in fungi, possibly being associated with their persistent life cycle that lacks an extracellular state (Nuss, 2005; Ghabrial et al., 2015). Accumulating evidence suggests that the capsidless lifestyles are diverse, while their details have been revealed for few viruses. The representative capsidless mycoviruses are members of the families *Hypoviridae*, *Endornaviridae*, and *Narnaviridae*, the families of positive-sense, single-stranded RNA [(+)RNA] viruses with non-segmented genome. Instead of capsids, the host membranous vesicles encapsulate their genomic RNAs, and this associations may protect the virus against antiviral defenses in the host fungi (Lakshman et al., 1998; Solorzano et al., 2000; Jacob-Wilk et al., 2006; Hillman and Cai, 2013; Suzuki et al., 2018; Valverde et al., 2019). The members of *Hypoviridae*, *Endornaviridae*, and *Narnaviridae* are phylogenetically distant (Wolf et al., 2018). Other non-segmented fungal RNA viruses that appear to have a potential capsidless nature, include unclassified viruses called yadokariviruses (Zhang et al., 2016; Hisano et al., 2018), fusariviruses (Kwon et al., 2007; Zhang et al., 2014), and phlegiviruses (Kozlakidis et al., 2009; Magae, 2012). Fusariviruses and phlegiviruses show moderate or low phylogenetic affinity to (+)RNA viruses of the family *Hypoviridae* or to double-stranded RNA (dsRNA) viruses of the family *Megabirnaviridae*, respectively (Petrzik et al., 2016; Suzuki et al., 2018; Sato et al., 2019). Their virus particles cannot be obtained by conventional purification methods (Kozlakidis et al., 2009; Magae, 2012; Zhang et al., 2014), and unstable particle-like forms have been reported for these viruses (Kwon et al., 2007). Thus, how fusariviruses and phlegiviruses protect their genome remains unknown. Unlike the aforementioned capsidless viruses, yadokariviruses (monopartite (+)RNA viruses), which show a distant phylogenetic relationship to some animal (+)RNA viruses such as caliciviruses (the picornavirus-like superfamily), hijack the capsids of other unrelated dsRNA viruses as their potential replication sites and behave like encapsidated dsRNA viruses (Zhang et al., 2016; Hisano et al., 2018).

Recent studies suggest that multi-segmented RNA viruses can also be capsidless, which has been shown for some polymycoviruses (Kanhayuwa et al., 2015; Zhai et al., 2016; Kotta-Loizou and Coutts, 2017; Niu et al., 2018) and one hadakavirus (Sato et al., 2020). Polymycoviruses are a group of mycoviruses belonging to the genus *Polymycovirus* in the family *Polymycoviridae*, which was established in 2020^1^. The genus accommodates six species with relatively characterized viruses and four species with uncharacterized viruses, for which only genomic information is available (top six and lower four, respectively, in Table 1). Like yadokariviruses, the type member of polymycovirus is distantly related to caliciviruses (Kanhayuwa et al., 2015). All known polymycoviruses share four conserved genomic segments, called dsRNA1, dsRNA2, dsRNA3, and dsRNA4, which encode P1 (RNA-dependent RNA polymerase, RdRP), P2 (hypothetical protein with unknown function, containing a transmembrane domain and a zinc-finger motif), P3 (putative methyltransferase, MTR), and P4 (a potential genome-associated protein named proline-alanine-serine-rich protein, PASrp), respectively (Kanhayuwa et al., 2015; Zhai et al., 2016; Jia et al., 2017; Kotta-Loizou and Coutts, 2017; Niu et al., 2018; Mahillon et al., 2019; Nerva et al., 2019; and unpublished sequence data deposited in DDBJ/EMBL/GenBank). Several polymycoviruses have additional one to four genomic segments, among which no significant sequence similarities were found (Zhai et al., 2016; Jia et al., 2017; Kotta-Loizou and Coutts, 2017; Mahillon et al., 2019). Hadakavirus is a novel (+)RNA virus that has a close phylogenetic relationship with polymycoviruses (Sato et al., 2020). In contrast to polymycoviruses, the hadakavirus has 11 dsRNA segments as its replicative form, which include three segments cognate with polymycovirus, namely -dsRNA1, -dsRNA2, and -dsRNA3 (Sato et al., 2020). Total genome size considerably varies between polymycoviruses and a hadakavirus due to the expanded genomic segment numbers (Table 1). The diversification of the genomic segment number among them may be related to their virus forms.

**TABLE 1:** Information of the 10 polymycoviruses and a closely related virus, HadV1.

Virus name		Genome information			Accession number for conserved proteins				References
Full	Abbreviation	Total size, kbp or kb	Segment number	Segment size range, kbp or kb	P1 (RdRP)	P2	P3 (MTR)	P4 (PASrp)	
Aspergillus fumigatus tetramycovirus 1	AfuTmV1	7.7	4	1.1−2.4	CDP74618	CDP74619	CDP74620	CDP74621	Kanhayuwa et al., 2015
Beauveria bassiana polymycovirus 1	BbPmV1	8.0	4	1.4−2.4	CUS18595	CUS18596	CUS18597	CUS18598	Kotta-Loizou and Coutts, 2017
Botryosphaeria dothidea RNA virus 1	BdRV1	8.7	5	1.1−2.4	AKE49495	AKE49496	AKE49497	AKE49498	Zhai et al., 2016
Colletotrichum camelliae filamentous virus 1	CcFV1	12.3	8	1.0−2.4	ASV63092	ASV63093	ASV63094	ASV63095	Jia et al., 2017
Fusarium redolens polymycovirus 1	FrPmV1	12.2	8	0.9−2.5	QDH44656	QDH44657	QDH44658	QDH44659	Mahillon et al., 2019
Penicillium digitatum polymycovirus 1	PdPmV1	8.0	4	1.3−2.4	AVZ65983	AVZ65984	AVZ65985	AVZ65986	Niu et al., 2018
Aspergillus spelaeus tetramycovirus 1	AspTmV1	7.8	4	1.2−2.4	AYP71805	AYP71808	AYP71807	AYP71806	Nerva et al., 2019
Cladosporium cladosporioides virus 1	CcV1	8.9	5	0.9−2.4	AII80567	AII80568	AII80569	AII80570	Unpublished
Magnaporthe oryzae polymycovirus 1	MoPmV1	7.9	4	1.3−2.4	QAU09249	QAU09250	QAU09251	QAU09252	Unpublished
Penicillium brevicompactum tetramycovirus 1	PbTmV1	7.8	4	1.2−2.4	AYP71801	AYP71804	AYP71803	AYP71802	Nerva et al., 2019
Hadaka virus 1	HadV1	15.3	11	0.9−2.5	BBU94038	BBU94039	BBU94040	Absent	Sato et al., 2020

Two viral forms have been proposed for polymycoviruses: a capsidless RNA-protein complex (RNP) form (Kanhayuwa et al., 2015; Zhai et al., 2016; Kotta-Loizou and Coutts, 2017; Niu et al., 2018) and a filamentous encapsidated form (Jia et al., 2017). The dsRNA viruses are generally icosahedral virions. The filamentous polymycovirus, named Colletotrichum camelliae filamentous virus 1 (CcFV1), was identified as the first and the only dsRNA virus with a filamentous virion architecture (Jia et al., 2017). In both capsidless and filamentous polymycoviruses, viral dsRNA was considered to be associated with virally encoded PASrp (Kanhayuwa et al., 2015; Zhai et al., 2016; Jia et al., 2017; Kotta-Loizou and Coutts, 2017; Niu et al., 2018). The capsidless form, suggested as colloidal, was first described for Aspergillus fumigatus tetramycovirus 1 (AfuTmV1), which was observed by atomic force microscopy (AFM) (Kanhayuwa et al., 2015). No typical virus particles have also been observed for Beauveria bassiana polymycovirus 1 (BbPmV1), Botryosphaeria dothidea RNA virus 1 (BdRV1), and Penicillium digitatum polymycovirus 1 (PdPmV1) by AFM or transmission electron microscopy (TEM) (Zhai et al., 2016; Kotta-Loizou and Coutts, 2017; Niu et al., 2018). The PASrp-associated dsRNA form, or even the purified dsRNA form, of some polymycoviruses are infectious when introduced into host fungal protoplasts (Kanhayuwa et al., 2015; Jia et al., 2017; Kotta-Loizou and Coutts, 2017; Niu et al., 2018). Thus, despite its capsidless nature, *Polymycoviridae* has been classified as a dsRNA virus family by the International Committee on Taxonomy of Viruses (ICTV). In contrast to polymycoviruses, the hadakavirus lacks PASrp or its related candidate and is hypothesized to exist as a soluble RNA form, although its alternative strategies for genome protection remain unknown (Sato et al., 2020). It should be noted that proline-alanine-serine (PAS)-rich proteins, phylogenetically unrelated to polymycovirus PASrps, are also encoded in other unclassified RNA viruses with a potentially capsidless nature, including phlegiviruses (Kozlakidis et al., 2009; Kanhayuwa et al., 2015; Kotta-Loizou and Coutts, 2017). Thus, it seems that PAS-rich proteins are required for certain RNA viruses. However, detailed molecular natures of the PASrps or other PAS-rich proteins, as well as the consensus PASrp-associated forms of polymycoviruses and other dsRNA viruses, remain obscure.

In this study, we report the molecular and biological characterization of a novel five-segmented polymycovirus, Penicillium janthinellum polymycovirus 1 (PjPmV1), which is a novel strain of the species *Penicillium digitatum polymycovirus 1* within the genus *Polymycovirus.* To gain further insights into the polymycovirus form, we present the relative buoyant density and sedimentation velocity of PjPmV1 as well as the nucleic acid binding capability of PjPmV1 PASrp.

## Materials and Methods

### Fungal Strains and Growth Conditions

The PjPmV1 was obtained from a fungal strain A58, which was collected in 2017 from a tobacco-potato double cropping soil in Manshera, Pakistan. Fungal species were identified by sequencing the amplified-internal transcribed spacer (ITS) region with the PCR primers (White et al., 1990) listed in Supplementary Table 1. Using BLASTN search, the ITS sequence of strain A58 was identical or most similar to that of *Penicillium janthinellum* (an ascomycete) (data not shown). The virus-free isogenic strains, including A58-cf1, were obtained by single conidium isolation from the original strain A58, which is infected by a novel polymycovirus PjPmV1. The presence or absence of PjPmV1 in conidial sub-isolates were initially determined by mycelial direct RT-PCR with PjPmV1-dsRNA1-specific primers (Supplementary Table 1) as previously described (Sato et al., 2020). The results were further confirmed by dsRNA extraction and by RT-PCR using total RNA templates. The PjPmV1-cured or PjPmV1-transmitted conidial sub-isolates were named “A58-cf*n*” or “A58-cv*n*,” respectively (different number *n* denotes independent sub-isolates).

Fusarium oxysporum chrysovirus 1 (FoCV1) strain A60, an unpublished multi-segmented dsRNA virus related to alphachrysoviruses, was used for virus purification analyses as a typical encapsidated virus. The FoCV1-infected *Fusarium oxysporum* (an ascomycete) strain A60 and its virus-free conidial sub-isolate A60-cf1 were used for this study.

Two virus-free ascomycetous fungi, *Rosellinia necatrix* strain W97 (Sasaki et al., 2007; Shimizu et al., 2018) and *Cryphonectria parasitica* strain EP155 (Crouch et al., 2020), were used for nucleic acid extraction (ribosomal RNA and genomic DNA, respectively) to analyze the interaction with recombinant PjPmV1-PASrp (see the section “Electrophoretic Mobility Shift Assay” in “Materials and Methods”).

The above fungal strains were grown on Difco potato dextrose agar (PDA) media (Becton, Dickinson and Co.) on a laboratory bench at room temperature. The fungal colonies were transferred to new plates before they covered the entire plate area. Conidia of *P. janthinellum* were naturally generated on PDA media.

### Extraction, Sequencing, and Northern Hybridization of dsRNA

The dsRNA was extracted from 3- or 5-day-old mycelia cultured on cellophane-PDA media and treated with RQ1 DNase (Promega Corp.) and S1 nuclease (Thermo Fisher Scientific, Inc.) as previously described (Sato et al., 2020). The purified dsRNA was separated on 1% (w/v) agarose gel in 0.5 × TBE. For the electrophoretic mobility shift assay (EMSA) described below, TAE was used instead of TBE. The dsRNA extracted from 125 mg (fresh weight) of mycelia (a starting volume) was loaded into a lane in the gel and stained with ethidium bromide (EtBr). The dsRNA band size was estimated based on the migration positions of mycoreovirus 1/S10ss (Sun and Suzuki, 2008). The dsRNA standards were purified from *C. parasitica* infected with the mycoreovirus 1/S10ss and was denoted as “M-dsRNA” in this paper.

The complete genome sequence of PjPmV1 was determined by next-generation sequencing (NGS) and Sanger sequencing. We performed NGS (HiSeq 2500, Illumina, Inc.) as previously described (Jamal et al., 2019; Shamsi et al., 2019). For NGS, we submitted a mixed dsRNA preparation (called “pool A1”) from three fungal strains including *P. janthinellum* strain A58 and *Alternaria alternata* strain A16 (Jamal et al., 2019). The 5’- and 3’-terminal nucleotide sequences were determined by 3’ RNA ligase-mediated rapid amplification of cDNA ends (3’ RLM-RACE) using dsRNA as templates with the method of Lin et al. (2013). Five to nine 3’ RLM-RACE clones for each terminus were analyzed. Primers used for 3’ RLM-RACE are listed in Supplementary Table 1. The viral sequence obtained by the NGS analysis was further confirmed by resequencing of cloned cDNAs synthesized by a PCR-based method. For RT-PCR in 3’ RLM-RACE and resequencing, Moloney murine leukemia virus (M-MLV) reverse transcriptase (Promega Corp.) and KOD -Plus- Neo DNA polymerase (Toyobo Co., Ltd.) were used. Hypothetical open reading frames (ORFs) were predicted by BLASTX^2^ and ORFfinder^3^. Conserved protein domains were detected by BLASTP search of the entire database (non-redundant protein sequences, nr) including CDD/SPARCLE (Lu et al., 2020).

Multiple sequence alignment of the 5’- or 3’-terminal nucleotide sequences of each genomic segment was conducted using ClustalW2 in GENETYX-MAC ver. 20.1.0 (GENETYX Corp.).

Northern hybridization of viral RNAs was also performed as previously described (Sato et al., 2020). Digoxigenin (DIG)-11-dUTP-labeled cDNA probes to PjPmV1-dsRNA segments were amplified with primers listed in Supplementary Table 1.

### RT-PCR With Total RNA Templates

Conidial sub-isolates of *P. janthinellum* A58 was checked by RT-PCR with total RNA templates for the presence of PjPmV1-genomic segments. Total RNA enriched with single-stranded RNA (ssRNA) was extracted from 3-day-old fungal colonies on cellophane-PDA by the method previously described for other fungi (Eusebio-Cope and Suzuki, 2015). Ten nanograms of RNA were subjected to RT-PCR in a 10 μL-reaction mix of PrimeScript One Step RT-PCR Kit Ver.2 (Takara Bio, Inc.). Host β-tubulin (*benA*) used as a control for RT-PCR was detected with the Bt2a and Bt2b primers (Glass and Donaldson, 1995). Used primers are listed in Supplementary Table 1.

### Phylogenetic Analysis

The phylogenetic relationships of PjPmV1 to 10 reported polymycoviruses (Table 1) were analyzed based on predicted amino acid sequences of four conserved proteins. The four proteins of polymycoviruses were described as PmV-P1 (putative RdRP), PmV-P2 (hypothetical protein with unknown function), PmV-P3 (putative MTR), and PmV-P4 (putative PASrp). Each cognate protein of HadV1 was employed as an outgroup, except for PmV-P4 missing from HadV1. The amino acid sequences were retrieved with accession numbers listed in Table 1. Multiple sequence alignment was performed by using online MAFFT ver. 7 with default settings (Katoh et al., 2019). Trees were constructed by the maximum likelihood (ML) method with best models in MEGA X with default settings (Kumar et al., 2018). The branch probabilities were analyzed by 500 times bootstrap resampling.

### Purification of Virus Particle (VP)-Like Forms

Virus particle (VP) or VP-like form (VPL) fractions of FoCV1 and PjPmV1 were obtained as follows. The crude VPL of PjPmV1 was obtained by two methods: with or without carbon tetrachloride (CCl_4_) clarification. In both cases, 5-to-7-day-old fungal colonies cultured on PDA-cellophane were used. Frozen fungal cultures were ground to powder in liquid nitrogen and then mixed with four volumes (v/w) of 0.1 M sodium phosphate (pH 7.0) containing 0.1% (v/v) of β-mercaptoethanol. For the method using CCl_4_, mycelial homogenates were clarified with 20% (v/v) of CCl_4_ and centrifuged at 2,000 × *g* for 20 min at 4°C. This step was repeated once. A small amount of CCl_4_ remained in the supernatants was evaporated in a desiccator by vacuum pumping for 10 min. For the method without CCl_4_, cell debris in mycelial homogenates was alternatively removed by centrifugation at 8,000 × *g* for 10 min at 4°C. In both cases, supernatants were ultracentrifuged at 130,000 × *g* for 1.5 h at 4°C in a 70Ti rotor by Optima L-100K (Beckman Coulter, Inc.). Pellets were resuspended in 0.1 volume (against the volume of the crude extract before ultracentrifugation) of 0.05 M sodium phosphate (pH 7.0). Suspensions were centrifuged at 8,000 × *g* for 10 min at 4°C. Supernatants were defined as “crude VP or VPL” and further subjected to cesium chloride (CsCl) or sucrose gradient centrifugation.

For CsCl gradient centrifugation, 2 mL each of 50, 40, 30, 20, and 10% (w/w) CsCl in 0.05 M sodium phosphate (pH 7.0) were overlaid from bottom to top and left overnight at room temperature. Subsequently, 2 mL of crude VP (VPL) suspensions were overlaid on the CsCl gradient and ultracentrifuged at ∼210,000 × *g* for 2 h at 16°C in an SW 41Ti rotor by Optima L-100K (Beckman Coulter, Inc.). For sucrose gradient centrifugation of VP (VPL) extracted with CCl_4_, 2 mL each of 50, 40, 30, 20, and 10% (w/v) sucrose in 0.05 M sodium phosphate (pH 7.0) were overlaid and left overnight at 4°C. For sucrose gradient centrifugation of VPL extracted without CCl_4_, 5 mL of 72%, 3 mL of 55%, and 2 mL of 10% (w/v) sucrose in 0.05 M sodium phosphate (pH 7.0) were overlaid and left overnight at 4°C. Two milliliter of crude VP (VPL) suspensions were overlaid on the sucrose gradients and ultracentrifuged at ∼210,000 × *g* for 2 h at 4°C in the same swing rotor. After gradient ultracentrifugation, all 1-mL fractions from top to bottom were collected and numbered from #1 to #12. Each fraction containing PjPmV1 dsRNA was mixed together and diluted with three volumes of 0.05 M sodium phosphate (pH 7.0) and ultracentrifuged at 130,000 × *g* for 1.5 h at 4°C in a 70Ti rotor by Optima L-100K (Beckman Coulter, Inc.). Pellets were resuspended in 30 μL of 0.05 M sodium phosphate (pH 7.0) and defined as “pure VPL.” Certain amounts of pure VPLs were filtered through a 0.2-μm membrane and used for dsRNA extraction, protein analyses, and transfection.

The pure VPLs were negatively stained with a fourfold diluted EM stainer (an alternative for uranyl acetate, Nissin EM Co.) and subsequently observed in a transmission electron microscope (TEM) Hitachi H-7650.

### Attempts at Virus Transfection

We tried to transfect pure VPLs and purified dsRNA of PjPmV1 into *P. janthinellum* strain A58-cf1, but the attempt was unsuccessful. Briefly, spheroplasts of A58-cf1 were prepared according to a standard method developed for *C. parasitica* (Churchill et al., 1990). Transfection was performed using a polyethylene glycol (PEG)-mediated method (Hillman et al., 2004), which was applied to a polymycovirus (Kanhayuwa et al., 2015). Subsequently, 100 μL of a suspension containing 1 × 10^7^ cells/mL spheroplasts was mixed with 10 μL of filtered pure VPL containing approximately 0.1 μg/μL of dsRNA. We used VPLs purified by two different ways: one extracted with CCl_4_ and separated by CsCl gradient centrifugation and the other one extracted without CCl_4_ and separated by sucrose gradient centrifugation. One hundred colonies were transferred from regeneration media to new PDA and screened for PjPmV1-dsRNA1 by mycelial direct RT-PCR. We also tried to transfect *P. janthinellum* spheroplasts (1 × 10^6^ cells) with purified dsRNA preparations (10 μL of approximately 0.5 μg/μL dsRNA) by the same method.

### Antibody Preparation and Immunoprecipitation

As antigens for the PjPmV1-PASrp (P4) antibody preparation, the recombinant proteins of PjPmV1-PASrp tagged with glutathione *S*-transferase (GST) at the N-terminal were expressed in *Escherichia coli*. The entire PjPmV1-PASrp ORF was amplified with primers listed in Supplementary Table 1 and cloned between the *Bam*HI and *Eco*RI sites of the pGEX-4T-3 vector (GE Healthcare, Ltd.) using an In-Fusion HD Cloning Kit (Takara Bio, Inc.). Cloned vectors were once transformed into *E. coli* strain DH5α to screen the proper construct. Plasmids were extracted using the GenElute^TM^ Plasmid Miniprep Kit (Sigma-Aldrich Co. LLC). The correct plasmid was transformed into *E. coli* strain BL21 (DE3) (New England Biolabs, Inc.) to purify the recombinant protein.

Recombinant protein (GST-PjPmV1-PASrp) was purified according to the manufacturer’s instruction (GE Healthcare, Ltd.). Briefly, expression of the recombinant protein gene in *E. coli* was induced with 0.6 mM isopropyl β-D-thiogalactopyranoside (IPTG) by shaking for 3 h at 37°C. Cultures were collected by centrifugation and resuspended into phosphate-buffered saline (PBS, pH 7.3). Resuspended cells were sonicated on ice and stirred with 1% (v/v) Triton X-100 at room temperature for 30 min. The solubilized proteins were purified with Glutathione Sepharose 4B (GE Healthcare, Ltd.) and GST-tag was not excised. These affinity-purified proteins were termed “native antigens.” Part of the affinity-purified proteins was separated by SDS−polyacrylamide gel electrophoresis (PAGE). A protein band of GST-PjPmV1-PASrp was excised and eluted by Model 422 Electro-Eluter (Bio-Rad Laboratories, Inc.). These proteins eluted after SDS−PAGE were called “denatured antigens.” The quality of the native and denatured antigens was checked by SDS−PAGE (Supplementary Figure 1). Antibody preparation was performed by Eurofins Genomics K.K. One milliliter of native and denatured antigens (more than 1 mg/mL concentration) was alternately injected into a New Zealand white rabbit five times at a 2-week interval.

Immunoprecipitation with anti-GST-PjPmV1-PASrp antibodies was performed as previously described (Zhang et al., 2016).

### SDS−PAGE and Western Blotting

Proteins extracted from mycelia or potential VP fractions (VPLs) were subjected to SDS−PAGE and western blotting. For extraction of mycelial proteins, frozen mycelia (5-day-old culture on PDA-cellophane) were ground in liquid nitrogen and suspended in four volumes (v/w) of PBS (pH 7.3). These mycelial extracts or pure VPLs (see above) were mixed with equal volumes of 2 × Laemmli sample buffer containing 12% (v/v) β -mercaptoethanol and denatured for 5 min at 100°C. The denatured proteins (10 μL for mycelial proteins and 2 μL for pure VPLs) were loaded in 10% (w/v) or 12% (w/v) polyacrylamide gel, and SDS−PAGE was performed according to a standard protocol (Sambrook and Russell, 2001). Total proteins were stained with Coomassie Brilliant Blue (CBB) R-250 using the Rapid Stain CBB Kit (Nacalai tesque, Inc.). Protein size was estimated with Precision Plus Protein Dual Color Standards (Bio-Rad Laboratories, Inc.) and denoted as “M-protein” in this paper.

Proteins on SDS−PAGE gels were transferred to a polyvinylidene difluoride (PVDF) membrane (Immobilon-P, Merck Millipore) in 10 mM *N*-cyclohexyl-3-aminopropanesulfonic acid (CAPS, pH 11.0) and 10% methanol. The membranes were first incubated with anti-GST-PjPmV1-PASrp antibody, followed by anti-rabbit IgG conjugated with alkaline phosphatase (formerly Kirkegaard & Perry Laboratories, Inc.) and nitro blue tetrazolium (NBT)-5-bromo-4-chloro-3-indolyl-phosphate (BCIP) solution, according to a standard protocol (Sambrook and Russell, 2001).

### Electrophoretic Mobility Shift Assay (EMSA)

The binding specificity of PjPmV1-PASrp with nucleic acids was analyzed by an electrophoretic mobility shift assay (EMSA). For this, GST-PjPmV1-PASrp and free GST (produced from pGEX-4T-3 empty vector) were purified by the method described above (see the section “Antibody Preparation and Immunoprecipitation”) with slight modifications. Briefly, glutathione sepharose beads were stringently washed prior to the elution steps. The beads were first washed three times in PBS (pH 7.3), as described in the normal protocol, and then four times with PBS containing 1 M NaCl, followed by three times with PBS. Approximately 1 mg/mL of GST-PjPmV1-PASrp and 4 mg/mL of GST were yielded in a maximum concentration. The protein concentration was estimated by standards of bovine serum albumin (BSA) on SDS−PAGE stained with CBB. The GST-PjPmV1-PASrp in part was heat-denatured for 5 min at 100°C. Purified proteins were incubated with 0.5−1.0 μg of dsRNA, ssRNA, or double-stranded DNA (dsDNA) in 50 mM Tris−HCl (pH 7.6), 150 mM NaCl, and 2.5 mM CaCl_2_ for 30 min at 30°C in a 10-μL mixture. The reaction solution (10 μL) was electrophoresed on 1.0% (w/v) agarose gel in 0.5 × TAE and stained with EtBr.

Nucleic acids used for EMSA were prepared as follows. PjPmV1-dsRNA was purified from mycelia of *P. janthinellum* strain A58 as described above, and FoCV1-dsRNA was purified from mycelia of *F. oxysporum* strain A60 by the same method. For the preparation of PjPmV1-RNA3 transcripts, a plasmid containing full-length cDNA to PjPmV1-dsRNA3 was prepared. Briefly, linear pGEM-T Easy vector (Promega Corp.) and PjPmV1-dsRNA3 were amplified with primers listed in Supplementary Table 1 and recombined with the In-Fusion HD Cloning Kit (Takara Bio, Inc.). The resulting plasmid was linearized by *Pst*I and used as a template for *in vitro* transcription by T7 RNA polymerase (Promega Corp.) according to the manufacturer’s instruction. Total RNA containing ribosomal RNA was extracted from *R. necatrix* strain W97 as previously described (Chiba et al., 2013). An expression cassette of hygromycin resistance gene (hygromycin B phosphotransferase, *HygR*) was amplified by PCR with primers listed in Supplementary Table 1, using pCPXHY3 (Guo et al., 2009) as a template. Genomic DNA was extracted from *C. parasitica* strain EP155 with phenol-chloroform-SDS followed by RNase A treatment.

## Results

### Molecular Characterization of PjPmV1

**FIGURE 1:**
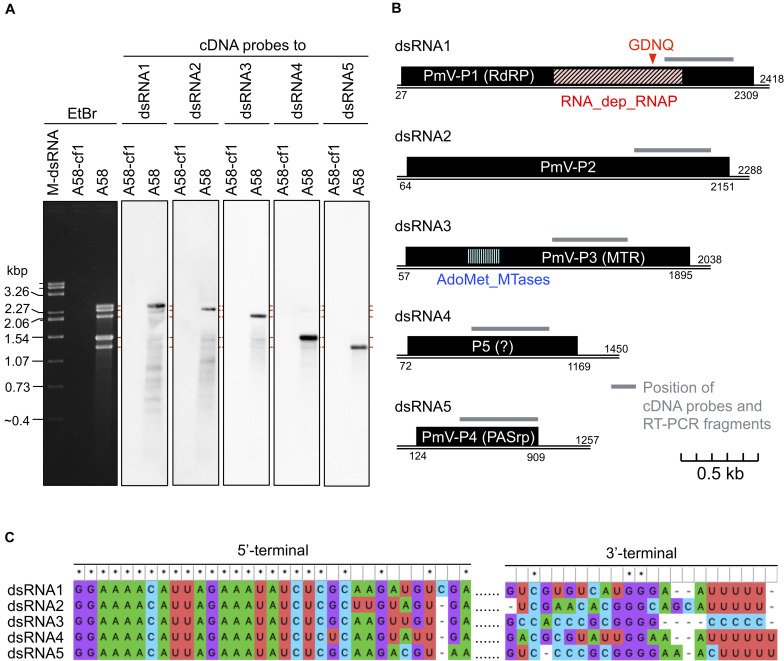
Genome organization of a novel polymycovirus PjPmV1. **(A)** Electrophoretic profile and northern hybridization of the viral dsRNA segments. *Penicillium janthinellum* A58 is the original strain infected with PjPmV1; A58-cf1 is a virus-free isogenic sub-isolate obtained from a single conidium of A58 (see Figure 3). Here and after “M-dsRNA” indicates the viral dsRNA (genomic segments of mycoreovirus 1/S10ss) as a molecular size maker. **(B)** Scheme of PjPmV1 genome organization. Black boxes indicate hypothetical open reading frames (ORFs). PmV-P1 (RdRP), -P2 (hypothetical protein), -P3 (MTR) and -P4 (PASrp) indicate ORFs encoding proteins homologous to polymyco-P1 (RNA-dependent RNA polymerase), -P2 (hypothetical protein with unknown function), -P3 (methyltransferase), or -P4 (proline-alanine-serine rich protein), respectively. RNA_dep_RNAP (RNA-dependent RNA polymerase domain, cd01699) and AdoMet_MTases (S-adenosylmethionine-dependent methyltransferases domain, cd02440) indicate positions of conserved protein domains hit in the CDD/SPARCLE database. An ORF shown on dsRNA4 is the hypothetical longest ORF predicted by ORFfinder. Gray bars indicate the position of cDNA probes (Figure 1A) and RT-PCR amplifications (Figure 3A and Supplementary Figure 3). **(C)** Comparison of 5’- and 3’-terminal nucleotide sequences among the five dsRNA segments. 5’- or 3’-terminal sequences were separately subjected to multiple sequence alignment. For analyses of the 5’-terminal, full sequences of each segment were aligned, and the 5’-terminal part is shown. For analyses of 3’-terminal, sequences of 3’-untranslated regions of each segment were aligned. The results were visualized in MEGA X.

A novel five-segmented polymycovirus, named Penicillium janthinellum polymycovirus 1 (PjPmV1), was discovered from one of fungal strains collected in Pakistan as previously described (Jamal et al., 2019; Shamsi et al., 2019; Sato et al., 2020). The host fungal strain A58 was identified as *Penicillium* species *P. janthinellum* based on the ITS sequence (see Materials and Methods). Five PjPmV1 dsRNA segments ranging approximately from 1.3 to 2.4 kbp, which was comparable to a size range of known polymycoviruses (Table 1), were accumulated in the strain A58 (Figure 1A). The complete genome sequence of the five dsRNA segments was obtained by NGS and Sanger sequencing (Figure 1B). The nucleotide sequences of the PjPmV1 genomic segments were deposited in EMBL/Genbank/DDBJ (accession numbers: LC571078-LC571082). These segments were numbered in decreasing order of the nucleotide length (Figure 1B, dsRNA1–5). Northern hybridization with segment-specific cDNA probes detected corresponding major single bands (Figure 1A). The BLASTX search revealed that the four genomic segments (dsRNA1, dsRNA2, dsRNA3, and dsRNA5) each encode proteins with 75–83% in amino acid identity to the counterparts of PdPmV1, a member of the genus *Polymycovirus* (Table 2). The PdPmV1 was isolated from a phytopathogenic *Penicillium* species, *P. digitatum*, and was reported to have a four-segmented dsRNA genome (Niu et al., 2018). The PjPmV1 dsRNA1, dsRNA2, dsRNA3, and dsRNA5 putatively encode proteins homologous to polymyco-P1 (RdRP), -P2 (hypothetical protein), -P3 (MTR), and -P4 (PASrp), respectively. The putative PjPmV1 RdRP contains “GDNQ” as the catalytic core residues which is common to known polymycoviruses and a hadakavirus as well as mononegaviruses (non-segmented, negative-stranded RNA viruses) (Kanhayuwa et al., 2015; Sato et al., 2020). The putative PjPmV1 PASrp contains 27% of PAS residues (data not shown), which is comparable to PASrps of other polymycoviruses containing 24–32% of PAS (Sato et al., 2020). The PjPmV1 dsRNA4 possesses an ORF (P5) encoding 365 amino acids (Figure 1B), which showed no significant similarity to any sequences by BLASTX and BLASTN search (Table 2 and data not shown).

**TABLE 2:** Summary of BLAST search with the PjPmV1 genomic segments.

Query	Hit*^*a*^*	Domain or motif (BLASTP search)	Top hit				
			Description	Query cover (%)	Identity (%)	*E*-value	Accession
dsRNA1	Yes	RNA_dep_RNAP (cd01699)	RNA-dependent RNA polymerase [Penicillium digitatum polymycoviruses 1]	94	75.30	0.0	YP_009551548.1
dsRNA2	Yes	None	hypothetical protein [Penicillium digitatum polymycoviruses 1]	91	78.42	0.0	YP_009551551.1
dsRNA3	Yes	AdoMet_MTases (cd02440)	methyltransferase [Penicillium digitatum polymycoviruses 1]	90	75.65	0.0	YP_009551549.1
dsRNA4	No	None	−	−	−	−	−
dsRNA5	Yes	None	hypothetical protein [Penicillium digitatum polymycoviruses 1]	62	82.76	1e−140	YP_009551550.1

*^a^Presence or absence of hits by BLASTX search of the database “nr, Non-redundant protein sequences”.*

The terminal nucleotide sequences of the five dsRNA segments of PjPmV1 were highly conserved (Figure 1C). The 5’-termini on the positive-sense strand of all dsRNA segments started with the 21-nt identical sequence (Figure 1C). The 3’-termini on positive sense of PjPmV1 dsRNA1, dsRNA2, dsRNA4, and dsRNA5 commonly stopped with three to eight “Us” (Figure 1C and Supplementary Figure 2A). By contrast, the 3’-terminus of PjPmV1 dsRNA3 stopped with five or six “Cs” (Figure 1C and Supplementary Figure 2A). The numbers of “U” (three to eight) or “C” (five or six) repeats varied among RACE clones (Supplementary Figure 2A). Figure 1 shows the majority sequence. The 19-nt of 5’-terminal nucleotide sequences are also common among all the genomic segments of PjPmV1 and PdPmV1 (Supplementary Figure 2B). By contrast, the 3’-terminal nucleotide sequences of PjPmV1 are distinct from that of PdPmV1, while those are conserved, to some extent, in each virus (Supplementary Figure 2C).

### Phylogenetic Analysis of PjPmV1

**FIGURE 2:**
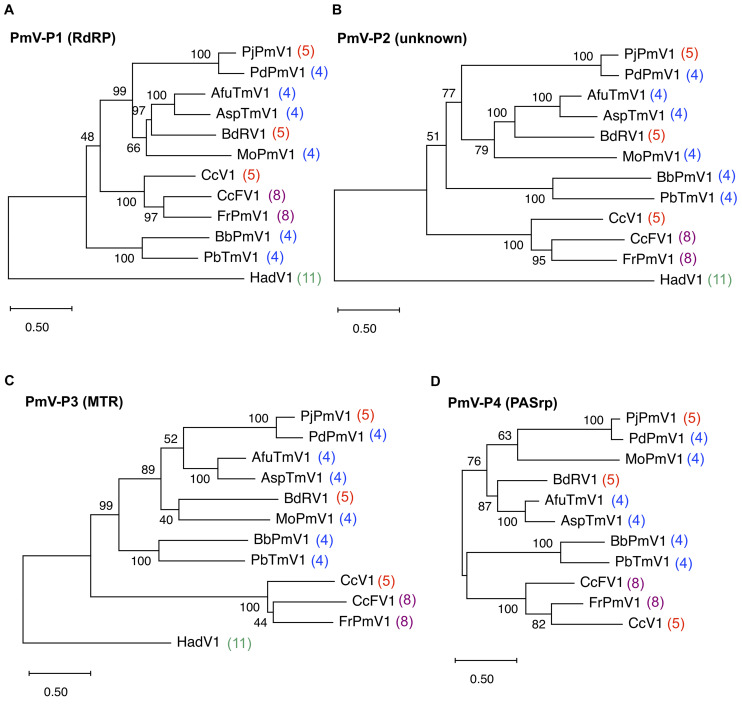
Maximum likelihood (ML) phylogenetic trees of PjPmV1 and other polymycoviruses. Trees were constructed based on amino acid sequence alignment of polymycovirus (PmV)-P1 **(A)**, -P2 **(B)**, -P3 **(C)**, or -P4 **(D)**. Analysis involved the members of 10 species in the family *Polymycoviridae* and one hadakavirus (an unassigned ssRNA virus) as an outgroup. For analysis of polymycovirus P4, no outgroup was employed. Abbreviations of virus names and accession numbers for each protein are listed in Table 1. Numbers behind the virus names indicate genomic segment numbers of each virus. The trees were constructed via the ML method with best fit models, namely LG + G + I + F for panels **(A–C)** and WAG + G + I for panel **(D)** in MEGA X. Scale bar and branch length indicate numbers of amino acid substitutions per site. Values next to the branches indicate a bootstrap probability in 500 iterations.

The phylogenetic relationships of PjPmV1 with 10 members of the genus *Polymycovirus* (Table 1) were analyzed based on the amino acid sequence of four conserved proteins using the ML method (Figure 2). The cognate proteins of a hadakavirus (HadV1) (Table 1) were employed as an outgroup except in the analysis of P4 (PASrp), which is absent in HadV1. When trees of different proteins were compared, their topology was largely similar, with a few minor differences in branching (Figures 2A–D). The PjPmV1 was most closely related to PdPmV1 in all the trees (Figures 2A–D), in accordance with the result of the BLASTX search (Table 2). The five-segmented polymycoviruses, namely PjPmV1, BdRV1, and CcV1, were placed to distinct branches (Figures 2A–D). The PjPmV1 and BdRV1 were distantly placed from eight-segmented polymycoviruses including CcFV1, the one with filamentous capsids (Figures 2A–D). Neither sequence similarity nor phylogenetic relationship was observed in their fifth to eighth segments (dsRNA5-dsRNA8), which are not conserved in the polymycoviruses (Supplementary Table 2). Thus, it seems that frequent gain or loss of specific genomic segments may spontaneously occur among polymycoviruses.

### Vertical Transmission of PjPmV1 to Conidia and Effects of PjPmV1 Infection on Host Fungal Growth

**FIGURE 3:**
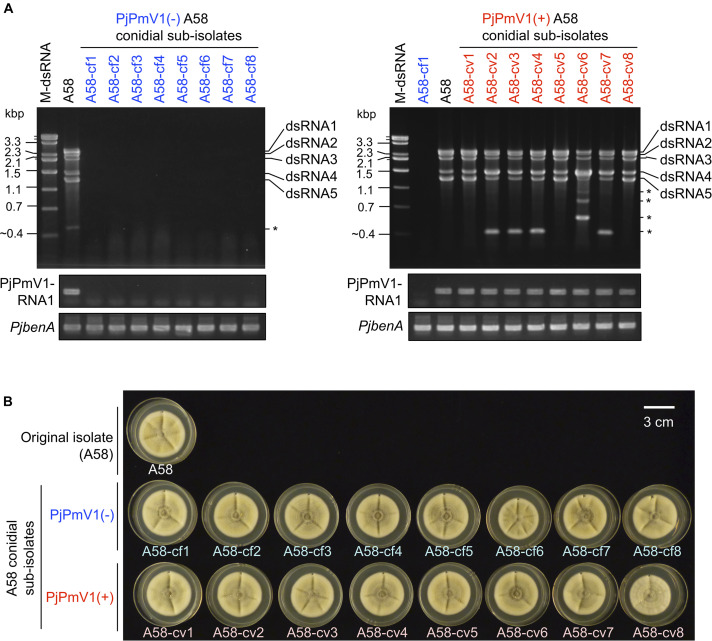
Vertical transmission of PjPmV1 to conidia. Eight independent sub-isolates were randomly selected from each PjPmV1-free [PjPmV1(–)] or PjPmV1-transmitted [PjPmV1(+)] population in conidial sub-isolates of *P. janthinellum* A58. **(A)** Detection of PjPmV1-derived RNAs in the eight PjPmV1(–) or PjPmV1(+) sub-isolates (left or right panels, respectively). The top panels show electrophoretic profiles of dsRNA-enriched fractions, while the middle and lower panels show RT-PCR products. Asterisks (*) indicate the position of putative defective or satellite-like dsRNAs. “M-dsRNA” indicates the molecular size of mycoreoviral dsRNAs. By RT-PCR, messenger RNAs of the RdRP-encoding dsRNA1 segment of PjPmV1 (PjPmV1-RNA1) were detected. Host β-tubulin (*benA*) gene was detected as a control for RT-PCR reaction. **(B)** Picture of fungal colonies (7-day-old) of *P. janthinellum* A58 sub-isolates cultured on PDA plates.

To analyze vertical transmission of PjPmV1 to conidia, we screened conidial sub-isolates of the original fungal strain A58 of *P. janthinellum*. We initially checked the presence or absence of PjPmV1 in 46 conidial sub-isolates based on the detection of the RdRP-encoding segment (PjPmV1-dsRNA1) by mycelial direct RT-PCR (data not shown). As a result, we obtained eight PjPmV1-free sub-isolates [PjPmV1(-), designated as A58-cf*n*] and remining PjPmV1-transmitted sub-isolates [PjPmV1(+), designated as A58-cv*n*]. No dsRNA bands were detectable in the eight PjPmV1(-) sub-isolates A58-cf1 to A58-cf8 (Figure 3A, top left panel). The RT-PCR detection of PjPmV1-dsRNA1 using total RNA preparations further confirmed that PjPmV1 was eliminated from all the PjPmV1(-) sub-isolates (Figure 3A, middle left panel). By contrast, all the PjPmV1 dsRNA segments were transmitted to the eight PjPmV1(+) sub-isolates A58-cv1 to A58-cv8, which were randomly selected from the 38 PjPmV1(+) sub-isolates (Figure 3A and Supplementary Figure 3). In several PjPmV1(+) conidial sub-isolates and the original strain A58, defective or satellite-like dsRNAs of various sizes occasionally appeared (indicated by an asterisk in Figure 3A). The presence of these defective species appeared to be associated with the ratio among PjPmV1 dsRNA segments, typically resulting in the decrease of relative dsRNA5 accumulation and the increase of dsRNA4 accumulation (Figure 3B).

We compared fungal growth between PjPmV1(-) and PjPmV1(+) sub-isolates on PDA media under laboratory conditions. As a result, no obvious growth change was observed depending on the presence or absence of PjPmV1 (Figure 3B and Supplementary Figure 4). Pigmentation accompanied with conidiation seemed different in some sub-cultures, but it was not correlated with the presence or absence of PjPmV1 (Supplementary Figures 4A,B). Average colony area tended to be slightly higher in PjPmV1(-) sub-isolates than in PjPmV1(+) sub-isolates, but the difference was neither statistically significant nor reproducible (Supplementary Figures 4C,D).

### CsCl and Sucrose Gradient Centrifugation of a Particle-Like Form of PjPmV1

**FIGURE 4:**
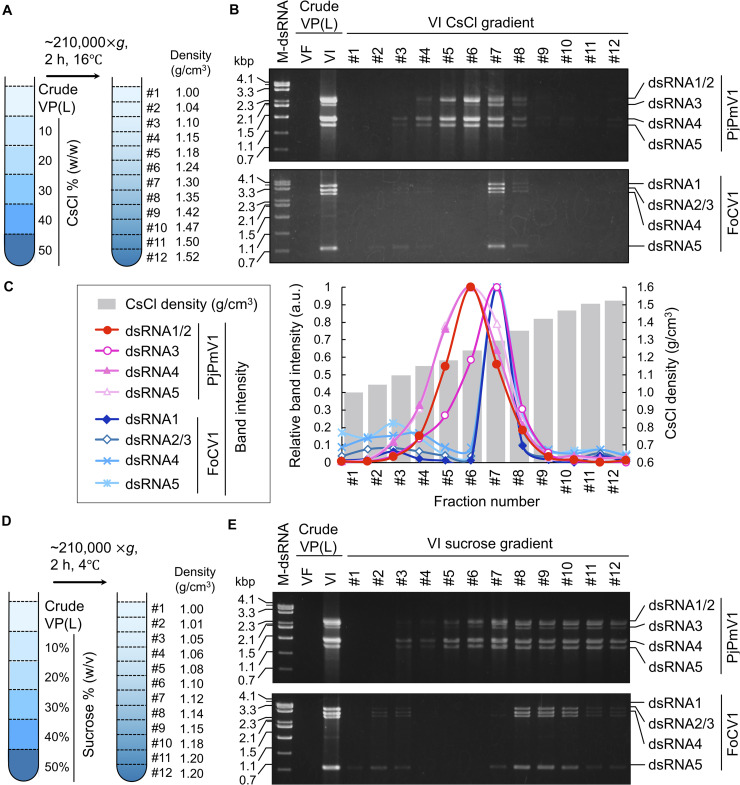
CsCl or sucrose density gradient centrifugation of PjPmV1 particle-like forms. **(A)** Scheme of the CsCl density gradient centrifugation. **(B)** Electrophoretic profile of viral dsRNA in the separated fractions after CsCl gradient centrifugation. **(C)** Digitalization of relative band intensity of panel **(B)** by Image J (https://imagej.nih.gov/ij/). Relative band intensity of every dsRNA segment is shown as a relative value against the peak value of each segment. **(D)** Scheme of sucrose density gradient centrifugation. **(E)** Electrophoretic profile of viral dsRNA in the separated fractions after sucrose gradient centrifugation. A Pakistani *Fusarium oxysporum* strain A60 infected by FoCV1 (an alphachrysovirus) was used as the reference of a typical encapsidated dsRNA virus. In panels **(B,E)**, “VF” and “VI” indicate virus-free or virus-infected fungal strains, respectively. In the analysis of PjPmV1 (top panel), “VI” or “VF” indicate *P. janthinellum* A58 or its conidial sub-isolate A58-cf1, respectively. In the analysis of FoCV1 (bottom panel), “VI” or “VF” indicate *F. oxysporum* A60 or its conidial sub-isolate A60-cf1, respectively. “M-dsRNA” indicates molecular size of dsRNA.

We have previously revealed that PjPmV1 dsRNA in mycelial homogenates is resistant to RNase A treatment (Sato et al., 2020), suggesting a protective form of the viral genomic dsRNA. Thus, we tried to analyze the physical nature of the protected dsRNA, assumed to be a potential virus particle-like form (VPL) (a capsidless RNP or filamentous particle). Our previous results suggest that this form can be extracted by an organic solvent (CCl_4_) and precipitated by ultracentrifugation (Sato et al., 2020). Thus, the VPL was first extracted with CCl_4_ and concentrated by ultracentrifugation. In parallel, we subjected FoCV1, an alphachrysovirus that makes rigid spherical capsids enclosing genomic dsRNA. The crude virus fractions of each virus were subjected to CsCl or sucrose density gradient centrifugation for 2 h (Figure 4). As a result of CsCl gradient centrifugation, PjPmV1 dsRNA was detected in much broader fractions than FoCV1-dsRNA (Figures 4A–C). That is, PjPmV1 dsRNA was detected in the fractions #4-#8 (1.15−1.35 g/cm^3^, and slightly in the fraction #3), while FoCV1 dsRNA was mainly detected in the fraction #7 (1.30 g/cm^3^, and slightly in the fractions #3 and #8) (Figures 4A–C). Interestingly, band intensity of PjPmV1 dsRNA3 peaked in the fraction #7, while intensity of the other segments peaked in the fraction #6 (Figures 4B,C). The broader fractionation of PjPmV1 was also observed by sucrose density gradient centrifugation (Figures 4D,E). In sucrose density gradient centrifugation, PjPmV1 dsRNA was detected in the fractions #5-#12 (1.08–1.20 g/cm^3^, and slightly in the fraction #3 and #4), while FoCV1 dsRNA was mainly detected in the fractions #8-#10 (1.14–1.18 g/cm^3^, and slightly in the other fractions) (Figures 4D,E). These results imply that potential PjPmV1 particle-like forms have uneven forms with various buoyant densities and sedimentation velocities.

To confirm that the above gradient centrifugation profiles were not caused by CCl_4_, we subsequently used crude VPLs obtained without CCl_4_. In gradient centrifugation, PjPmV1-dsRNA was also detected in the broad fractions (1.13–1.33 g/cm^3^ in the CsCl gradient and 1.09–1.23 g/cm^3^ in the sucrose gradient) (Supplementary Figures 5A-D). Thus, uneven PjPmV1 forms seemed to naturally occur in infected cells rather than being generated by CCl_4_.

### Components of a Particle-Like Form of PjPmV1

**FIGURE 5:**
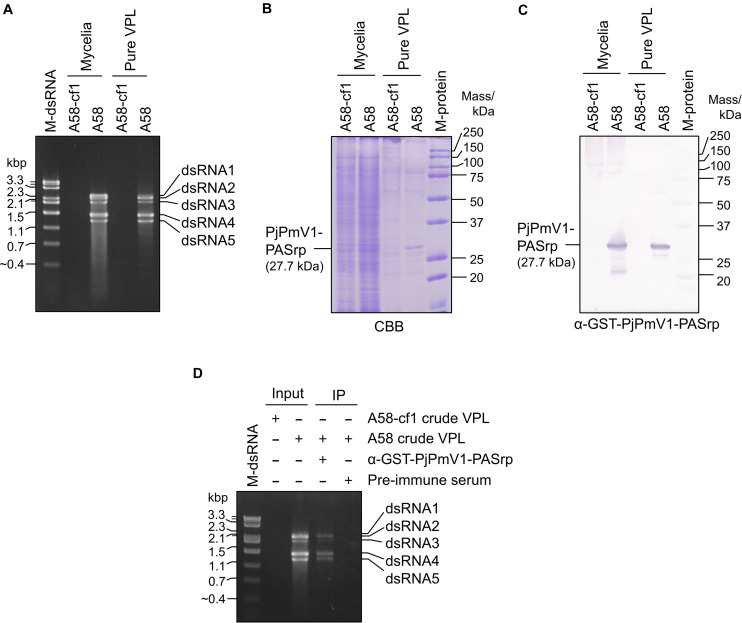
Components of a potential particle-like form of PjPmV1. **(A)** DsRNA extracted from mycelia or pure VPL (virus particle-like form) fraction. “Pure VPL” means resuspended pellets obtained by ultracentrifugation of the CsCl gradient fractions #3–#8 in Figures 4A–C. “M-dsRNA” indicates the molecular size of mycoviral dsRNA segments. **(B)** Detection of total proteins from mycelia or pure VPL on SDS–PAGE gel stained with CBB. **(C)** Detection of PjPmV1-PASrp (P4) in the total protein fractions from mycelia or pure VPL preparation using polyclonal antibody (α-GST-PjPmV1-PASrp) by western blotting. In panels **(B,C)**, proteins were separated on 12% (w/v) polyacrylamide gel. “M-protein” indicates the molecular size of proteins. **(D)** Immunoprecipitation of PjPmV1 particle-like forms in the crude VPL fraction with α-GST-PjPmV1-PASrp antibody. Electrophoretic profiles of dsRNA in input and immunoprecipitated (IP) fractions were shown. Pre-immune serum was used as control for IP.

To examine components of the potential PjPmV1 particle-like forms, we concentrated the fractions containing PjPmV1 dsRNA (fractions #3-#8 in Figure 4B) by further ultracentrifugation. The resuspended fraction, called “pure VPL,” retained all the dsRNA segments of PjPmV1 (Figure 5A). Detection of total proteins by SDS−PAGE revealed that the pure VPL from A58, but not from A58-cf1, contained a specific major band between 25 and 37 kDa, comparable to the expected molecular size of PjPmV1-PASrp (27.7 kDa) (Figure 5B). Immunological detection of PjPmV1 PASrp with polyclonal antibodies further confirmed that the major specific band corresponded to PASrp (Figure 5C). In contrast to A58, no signals were immunologically detected in mycelial proteins and pure VPL fractions from PjPmV1-free A58-cf1 (Figure 5C). In immunoprecipitation, PjPmV1-dsRNA was specifically co-purified with PjPmV1 PASrp antibody (Figure 5D). These results suggest that like other previously characterized polymycoviruses, PjPmV1 dsRNA was also associated with PASrp, which was the major component of the potential particle-like forms. However, we failed to detect any PjPmV1 particles with filamentous or icosahedral structure in any VPL preparations under TEM observation (data not shown), suggesting that PASrp-associated PjPmV1 dsRNA forms a capsidless RNP structure, as previously proposed.

We tried to transfect PjPmV1 using its pure VPL preparations, but this was unsuccessful. For the transfection, we used pure VPLs obtained by two ways: one extracted with CCl_4_ and separated by a CsCl gradient (Figures 4A–C, 5A–C) and the other extracted without CCl_4_ and separated by sucrose gradient centrifugation (Supplementary Figures 5C–E). The latter preparation was obtained by the mildest class of purification way. Approximately 100 colonies from each transfection tested negative. The attempts to transfect with purified dsRNA of PjPmV1 were also unsuccessful.

### Non-specific Interactions of PjPmV1-PASrp With Nucleic Acids

**FIGURE 6:**
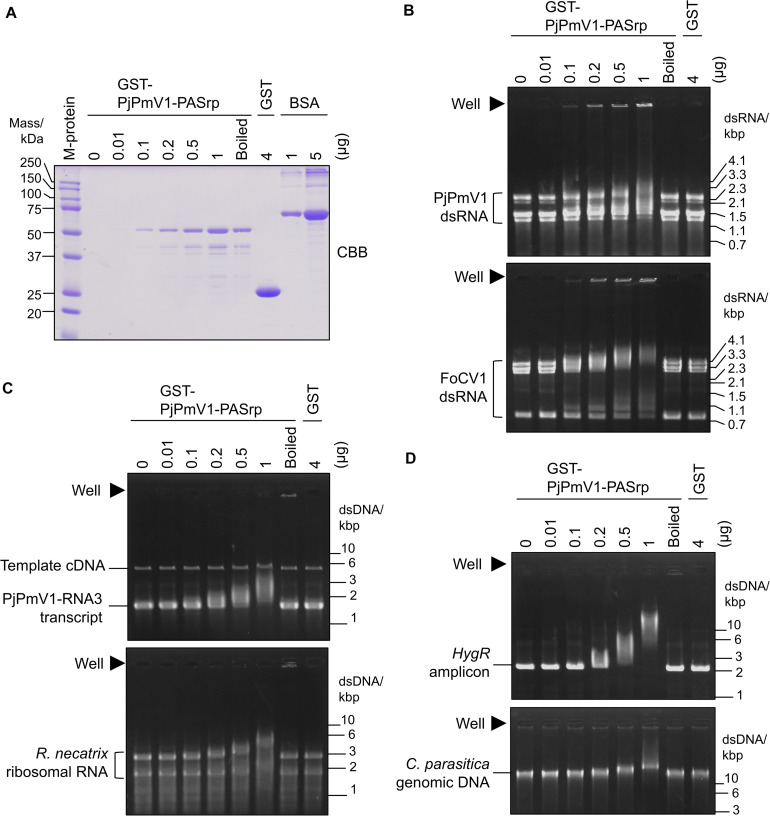
Nucleic acid interactions of PjPmV1-PASrp. Electrophoretic mobility shift assay (EMSA) of PjPmV1-PASrp was conducted with various nucleic acids. Various amounts of GST-PjPmV1-PASrp [0.01–1 μg (0.19–19 pmol) per lane] and a constant amount of GST [4 μg (0.15 nmol) per lane] were used. “Boiled” indicates heat-denatured GST-PjPmV1-PASrp (1 μg/μL of the protein was boiled and 1 μL per lane was used for each experiment). **(A)** CBB staining of purified recombinant proteins (GST-PjPmV1-PASrp and free-GST) and bovine serum albumin (BSA) in SDS–PAGE. BSA was used to estimate the amount of recombinant proteins. Proteins were separated on 12% (w/v) polyacrylamide gel. “M-protein” indicates size marker proteins with their respective molecular weight (kDa). **(B)** Interactions of PASrp with viral dsRNAs. PjPmV1 dsRNA from *P. janthinellum* A58 [upper panel, 1 μg (∼0.16 pmol) per lane], and FoCV1 dsRNA from *F. oxysporum* A60 [lower panel, 1 μg (∼0.12 pmol) per lane] were used. Here approximate total moles were calculated on the assumption that each dsRNA segment was accumulated at equal moles. **(C)** Interaction of PASrp with viral or non-viral ssRNAs. PjPmV1-RNA3 *in vitro* transcripts with its template cDNA [upper panel, 1 μg (1.5 pmol) per lane] and the total RNA extracted from *Rosellinia necatrix* (lower panel, 1 μg per lane) were used. **(D)** Interaction of PASrp with dsDNA. PCR amplicon of hygromycin resistance gene [*HygR* amplicon, upper panel, 0.5 μg (0.38 pmol) per lane] and genomic DNA extracted from *Cryphonectria parasitica* [lower panel, 0.5 μg (0.018 fmol) per lane] were used. In panel **(B)**, the size marker of dsRNA (from mycoreovirus 1/S10ss) was loaded in a lane of the same gels (not shown). In panels **(C,D)**, the size marker of dsDNA [GeneRuler 1 kb DNA ladder (Thermo Fischer Scientific, Inc.)] was loaded in a lane of the same gels (not shown). In panels **(B–D)**, filled triangles indicate position of wells.

While co-purification of polymyco-PASrps with their genomic dsRNA has previously been demonstrated, the binding properties of PASrps-nucleic acids have not been investigated. Thus, we examined whether PjPmV1-PASrp can interact with other nucleic acids besides its dsRNA genome by EMSA (an assay with gel electrophoretic mobility shift). We used various amounts (0.01–1 μg) of the recombinant GST-PjPmV1-PASrps including heat-denatured (boiled) ones and 4 μg of free-GST as a control (Figure 6A). The GST-PjPmV1-PASrp was more insoluble and yielded lower amounts than GST in *E. coli* (Figure 6A). These recombinant proteins were incubated with various dsRNA (viral dsRNA from PjPmV1 or FoCV1, Figure 6B), ssRNA (*in vitro* transcript of PjPmV1-RNA3 or ribosomal RNA from *R. necatrix*, Figure 6C), and dsDNA (PCR amplicon of hygromycin resistance gene from a plasmid vector or genomic DNA from *C. parasitica*, Figure 6D). The electrophoretic mobility of all nucleic acids became retarded by pre-incubation with GST-PjPmV1-PASrp in a dose-dependent manner (Figures 6B–D). Some of the dsRNA, but not ssRNA and dsDNA, incubated with native GST-PjPmV1-PASrp stayed in the wells on the gel (Figure 6B), suggesting stronger interaction of PASrp with dsRNA than with ssRNA or dsDNA. Simultaneously, no mobility shift was observed in samples pre-incubated with boiled GST-PjPmV1-PASrp or with excessive amounts of GST (Figures 6B–D).

Taken together, PjPmV1-PASrp of native conformation can interact with dsRNA, ssRNA, and dsDNA in a sequence-non-specific manner.

## Discussion

While polymycoviruses, a relatively newly established group, are classified as a dsRNA virus, they represent an evolutionary link between ssRNA and dsRNA viruses (Kanhayuwa et al., 2015; Sato et al., 2020). Here, we revealed the genome organization and detailed molecular characteristics of PjPmV1, which is the first polymycovirus isolated from *P. janthinellum* (Table 1). The PjPmV1 has a five-segmented dsRNA genome which consists of four conserved polymycoviral segments and one unique segment, showing no similarity to any known sequences (Figure 1 and Table 2). The PjPmV1 is most closely related to PdPmV1, a four-segmented polymycovirus from *P. digitatum*, rather than other five-segmented polymycoviruses (Figure 2). The five dsRNA segments share highly conserved terminal sequences (Figure 1C) and were transmitted to conidia in an all-or-none fashion (Figure 3A), which supports that all the five dsRNA species (dsRNA1-dsRNA5) were derived from PjPmV1.

The PjPmV1 dsRNA was associated with PASrp *in vivo* (Figure 5), similar to other polymycoviruses (Kanhayuwa et al., 2015; Zhai et al., 2016; Kotta-Loizou and Coutts, 2017; Niu et al., 2018). The PASrp-associated PjPmV1 dsRNA showed a broader range of buoyant density and sedimentation velocity than icosahedral virions of a chryovirus (a multi-segmented dsRNA virus) (Figure 4 and Supplementary Figure 5). The TEM observation of the purified PjPmV1 fractions revealed that the PASrp-associated dsRNA may not form filamentous or icosahedral structures (data not shown). Thus, PjPmV1-genomic dsRNA seems to exist as a non-rigid or unstable nucleoprotein form rather than as encapsidated form. This broader range of buoyant density and sedimentation velocity appears not to result from differently sized genomic segments, because of no great variation in segment ratios in different fractions (Figures 4B,E). The separate existence of the PASrp-associated dsRNA segments is suggested by the observation that the accumulation ratio of each dsRNA segment was unequal depending on sub-cultures. For example, dsRNA4 sometimes accumulated more than dsRNA5 (Figure 1) and *vice versa* (Figure 3A). These characteristics of the PASrp-associated forms might have contributed to the frequent gain or loss of specific genomic segments of polymycoviruses. The PjPmV1-PASrps interacted with various nucleic acids in a sequence-non-specific manner *in vitro* (Figure 6). The phosphoprotein of rhabdovirus, a member of the order *Mononegavirales*, has also non-specific nucleic acid-binding capability, and the rhabdovirus nucleoprotein serves as a chaperon to facilitate its specific binding with the viral genomic RNA (Masters and Banerjee, 1988; Mavrakis et al., 2006). There must be a mechanism by which PASrps is associated specifically with polymycovirus genomic dsRNA segments in cellular environments rich in diverse nucleic acids.

So far, we failed to transfect PjPmV1 in both PASrp-associated and purified dsRNA (PASrp-free) forms despite repeated attempts (data not shown), likely due to technical difficulty. Jia et al. reported that CcFV1 was also not infectious as a PASrp-associated form (filamentous particles), although it was infectious as purified PASrp-free dsRNA at low efficiency (approximately 2%) (Jia et al., 2017). Niu et al. also suggested that purified PASrp-free dsRNA of PdPmV1 was infectious at low efficiency (approximately 2–3%) (Niu et al., 2018), although they did not describe the transfection efficiency of the PASrp-associated form. Thus, the role of the PASrp-associated form of polymycoviral dsRNA in infection remains unclear. We showed, however, that PjPmV1 dsRNA associated with PASrp was tolerant to a ribonuclease (Sato et al., 2020), and its PASrp associated form was stable under organic solvent (CCl_4_) treatment (Figures 4, 5). Thus, an assumed role of PASrp is to protect the genomic RNA, as in the case of viral capsids.

According to the proposal for the genus *Polymycovirus* in the ICTV, species differentiation should be based on host fungus, identity of RdRP (≤70% in amino acid sequence), size and number of dsRNA segments, and presence of true capsid. The amino acid sequence of RdRP of PjPmV1 was 75% identical to that of PdPmV1 (Table 2), slightly higher than the species criteria. The genomic segments of both viruses share an identical 19-bp nucleotide sequence at 5’-terminal (Supplementary Figure 2B). On the other hand, both viruses have apparently different nucleotide sequences at the 3’-terminal (Supplementary Figure 2C). Furthermore, PjPmV1 has a five-segmented genome (Figure 1B), while PdPmV1 has only a four-segmented genome. The PdPmV1 was alternatively co-infected with a narna-like virus of 1702 nt (Niu et al., 2018), a similar size to the PjPmV1-specific segment (PjPmV1-dsRNA4, 1450-bp). Typical virus particles were neither observed for PdPmV1 (Niu et al., 2018) nor for PjPmV1 (data not shown); PjPmV1 and PdPmV1 were commonly isolated from fungi of same genus, *Penicillium*, but these hosts belong to two different species, *P. janthinellum* and *P. digitatum*, respectively. Taken together, PjPmV1 can be regarded as a strain distinct from PdPmV1, both of which belong to the same species, *Penicillium digitatum polymycovirus 1*.

Host phenotypic alterations are noted for a few polymycoviruses. For example, PdPmV1 along with a co-infecting narna-like virus increase fungicide susceptibility of *P. digitatum* and decreases virulence of the host to citrus fruits (Niu et al., 2018). However, it remains to be determined whether PdPmV1 or the narna-like virus is the major contributor to the phenotypic change. AfuTmV1 and CcFV1 were shown to confer mild hypovirulence in their fungal hosts (Kanhayuwa et al., 2015; Jia et al., 2017). Another previous study showed BdRV1 to induce hypovirulence in its phytopathogenic host *Botryosphaeria dothidea* (Zhai et al., 2016). In our case, PjPmV1 had no obvious effect on host growth under normal conditions, based on the macroscopic observation of conidial sub-isolates (Figure 3C and Supplementary Figure 4). However, we could not efficiently investigate the effects of PjPmV1 on the host because we failed to re-inoculate PjPmV1. In addition to the transfection failure, we could not inoculate PjPmV1 via hyphal anastomosis (data not shown). Further studies are therefore needed to identify infectious entities and inoculation ways of PjPmV1.

## Data Availability Statement

The datasets presented in this study can be found in online repositories. The names of the repository/repositories and accession number(s) can be found in the article/Supplementary Material.

## Author Contributions

NS and YS designed the experiments and wrote the manuscript. YS, AJ, and HK performed the experimental work. All authors analyzed the data and have given approval to the final version of the manuscript.

## Conflict of Interest

The authors declare that the research was conducted in the absence of any commercial or financial relationships that could be construed as a potential conflict of interest.

## Footnotes

1 https://talk.ictvonline.org/taxonomy/

2 https://blast.ncbi.nlm.nih.gov/Blast.cgi

3 https://www.ncbi.nlm.nih.gov/orffinder/
