# Supplementary material for: Molecular Characterization of a Novel Polymycovirus From Penicillium janthinellum With a Focus on Its Genome-Associated PASrp
Source: Front Microbiol. 2020 Oct 20;11:592789. doi: 10.3389/fmicb.2020.592789 (PMC7606342; doi:10.3389/fmicb.2020.592789)
Supplement: Supplementary file 1 [file Data_Sheet_1.pdf]

Supplementary information for

Molecular characterization of a novel polymycovirus from *Penicillium janthinellum* with a focus on its genome-associated PASrp

Yukiyo Sato[1], Atif Jamal[2], Hideki Kondo[1], Nobuhiro Suzuki[1]

[1] Institute of Plant Science and Resources, Okayama University, Kurashiki, Japan

[2]Crop Diseases Research Institute (CDRI), National Agricultural Research Centre (NARC), Islamabad, 45500, Pakistan

Running title: **Characterization of the genome and PASrp of a novel polymycovirus, PjPmV1**

* Corresponding Author: Agrivirology Laboratory
Institute of Plant Science and Resources
Okayama University
Kurashiki, Okayama 710-0046, Japan
Tel. 81(86) 434-1230
Fax. 81(86) 434-1232
e-mail. nsuzuki@okayama-u.ac.jp

Supplementary Figure S1. Primers used in this study

For	Target	Primer sequence (5'-3')	
		Forward	Reverse
Fungal identification	Fungal ITS	TCCGTAGGTGAACCTGCGG (ITS1)	TCCTCCGCTTATTGATATGC (ITS4)
Fungal identification and RT-PCR	*P. janthinellum benA*	GGTAACCAAATCGGTGCTGCTTTC (Bt2a)	ACCCTCAGTGTAGTGACCCTTGGC (Bt2b)
3' RLM-RACE[a]	5'-terminal of PjPmV1-dsRNA1	-	GGAGGTTGCGTGCAGCATAA
	3'-terminal of PjPmV1-dsRNA1	ATGGGCGTTTTGACGGACTA	-
	5'-terminal of PjPmV1-dsRNA2	-	TTGTAGAGTGGGATCCGGAA
	3'-terminal of PjPmV1-dsRNA2	CCAGACGTTACGGTGCTAAC	-
	5'-terminal of PjPmV1-dsRNA3	-	TCCACCCGAGCTAACAGAAA
	3'-terminal of PjPmV1-dsRNA3	AGACCAAGAGTTGCCAGGTG	-
	5'-terminal of PjPmV1-dsRNA4	-	GCCACAATCACCCACAATGT
	3'-terminal of PjPmV1-dsRNA4	TCAACTTGAGGGTGTTCCAG	-
	5'-terminal of PjPmV1-dsRNA5	-	GAAACCAAACGTGGAACCGA
	3'-terminal of PjPmV1-dsRNA5	AAGCACCGACCTCATGATCG	-
DIG-labelling PCR and/or RT-PCR	PjPmV1-dsRNA1 (1738-2186 nt)	ACGAGACGGAAGTGTCCAAC	TAGCAATGCCGTTCCGAGTT
	PjPmV1-dsRNA2 (1541-2040 nt)	CTACTGGACCTGGCACCATA	AAACTCACGATGGTAGGGGT
	PjPmV1-dsRNA3 (1001-1490 nt)	GTCGTCCACTCACGTTCTTG	GCCACGTATGTACCTCAGGA
	PjPmV1-dsRNA4 (0481-0985 nt)	ACAGCGTGTCTATTGCTGGA	TGAAGATCCCTTGGAGTCAG
	PjPmV1-dsRNA5 (0401-0909 nt)	ACGCGCTGGAGATCTTAAAG	agtcgacccgggaattcTCAGCCGACTTTGCTGGTAC
Vector construction[a]	PjPmV1-PASrp ORF	ggttccgcgtggatccATGTCGACACCCTCGGTTCC	agtcgacccgggaattcTCAGCCGACTTTGCTGGTAC
	pGEM-T	CTATAGTGTCACCTAAATAGCTTG	TATAGTGAGTCGTATTACAATTCACT
	PjPmV1-dsRNA3 (full length)	atacgactcactataGGAAAACATTAGAAATATCTC	taggtgacactatagCTGCAGGGGGGCCCCGCGGGTGGCAA
PCR	Hygromycin resistance gene cassette on pCPXHY3	CAGAAGATGATATTGAAGGAGCA	TCTAGAAAGAAGGATTACCTCTAA

[a] Primers for 3' RLM-RACE to identify the 5'- and 3'-terminal nucleotide sequences on the positive strands of PjPmV1-dsRNA segments.
[b] Underlined nucleotides are overlapped sequence between linearized vectors and inserts for In-Fusion cloning.

Supplementary Table S2. List of non-conserved polymycovirus genomic segments

Virus name		Specific genomic segments		Hit[a]	
**Full**	**Abbreviation**	**Segment number**	**Accession No.**	**BLASTN (megablast)**	**BLASTX**
Botryosphaeria dothidea RNA virus 1	BdRV1	dsRNA5	KP245738	None	None
Colletotrichum camelliae filamentous virus 1	CcFV1	dsRNA5	KX778770	None	None
		dsRNA6	KX778771	None	None
		dsRNA7	KX778772	None	None
		dsRNA8	KX778773	None	None
Fusarium redolens polymycovirus 1	FrPmV1	dsRNA5	MK609924	None	None
		dsRNA6	MK609925	None	None
		dsRNA7	MK609926	None	None
		dsRNA8	MK609927	None	None
Cladosporium cladosporioides virus 1	CcV1	dsRNA5	KJ787690	None	None

[a] Hits detected by BLASTN (megablast) search of the database "Nucleotide collection (nr/nt)" or BLASTX search of the database "Non-redundant protein sequences (nr)". Hits to sequences of their own and of the same polymycovirus species (different isolates) were excluded.

Supplementary Figure S1. Antigens for the preparation of antibodies against recombinant PjPmV1-PASrp (P4). Native (N*, approximately 3 μg) and denatured (D*, approximately 1 μg) GST-PjPmV1-PASrps preparations were subjected to SDS-PAGE. Native means proteins just purified with glutathione sepharose. Denatured means proteins eluted from a major band on SDS-PAGE of native proteins. 10% (w/v) polyacrylamide gel was used. M-protein indicates molecular size of marker proteins.

Supplementary Figure S2. 5′- and 3′-terminal nucleotide sequence of PjPmV1 and PdPmV1 genome segments. (A) Percentage of RACE clones with different repeat numbers of "U" or "C" at 3′-terminal on positive sense of PjPmV1 segments. (B) Comparison of 5′-terminal nucleotide sequence between PjPmV1 and PdPmV1 segments. All genomic segments of PjPmV1 and PdPmV1 were together subjected to multiple sequence alignment, and the result of the 5′-terminal part is shown. (C) Comparison of 3′-terminal nucleotide sequence between PjPmV1 and PdPmV1 segments. 3′-UTR of each PjPmV1 or PdPmV1 segments were separately subjected to multiple sequence alignment. In (B) and (C), the result of sequence alignment was visualized in MEGA X. Each mark (spade, heart, club, diamond) in the left side indicates a pair of genomic segments conserved between PjPmV1 and PdPmV1.

Supplementary Figure S3. Vertical transmission of PjPmV1-dsRNA segments. The simultaneous transmission of PjPmV1-dsRNAs was confirmed in the eight PjPmV1(+) conidial sub-isolates of A58 by RT-PCR detection of PjPmV1mRNAs (-RNA2, -RNA3, -RNA4 and -RNA5) with purified total RNA templates. RNAs from PjPmV1-dsRNA1 and a host gene were detected in Fig. 3A.

Supplementary Figure S4. Comparison of host growth between PjPmV1-free [PjPmV1(-)] and PjPmV1-infected [PjPmV1(+)] conidial sub-isolates. Fungal sub-isolates are identical to those used in Fig. 3. (A) Front side picture of fungal colonies (14 days old) on PDA plates (9 cm in diameter). Subcultures independent from those in Fig. 3B were used. (B) Back side picture of fungal colonies used in (A). In (A) and (B), bars indicate 3 cm. (C) Quantification of colony area of the 7-day-old cultures in Fig. 3B. (D) Quantification of colony area of the 14-day-old cultures in Fig. S4A. In (C) and (D), quantification was

carried out with Image J (https://imagej.nih.gov/ij/). Mean values of each PjPmV1(-) or PjPmV1(+) (including A58) population are shown by bar graph. A dot indicates the value of each fungal colony. Statistical differences between PjPmV1(-) and PjPmV1(+) populations were analyzed by Welch's *t*-test in R version 3.5.2 (https://www.r-project.org). NS means no significant differences ($n = 8$ or 9, $p < 0.05$).

Supplementary Figure S5. CsCl or sucrose density gradient centrifugation of PjPmV1 particle-like forms extracted without organic solvents. (A) Scheme of the CsCl density gradient centrifugation. (B) Electrophoretic profile of PjPmV1-dsRNA segments in the separated fractions after CsCl centrifugation. (C) Scheme of sucrose density gradient centrifugation. (D) Electrophoretic profile of PjPmV1-dsRNA segments in the separated fractions after sucrose centrifugation. (E) Check of PjPmV1-dsRNA presence in pure VPL fractions. The pure VPL fraction was obtained by concentration of the sucrose gradient fractions #3–#7 in (D) and used for TEM observation and transfection trials. DsRNAs extracted from mycelia and crude VPL preparations were subjected as controls. "M-dsRNA" indicates molecular size marker of dsRNA (mycoreovirus 1/S10ss).

FIG S1

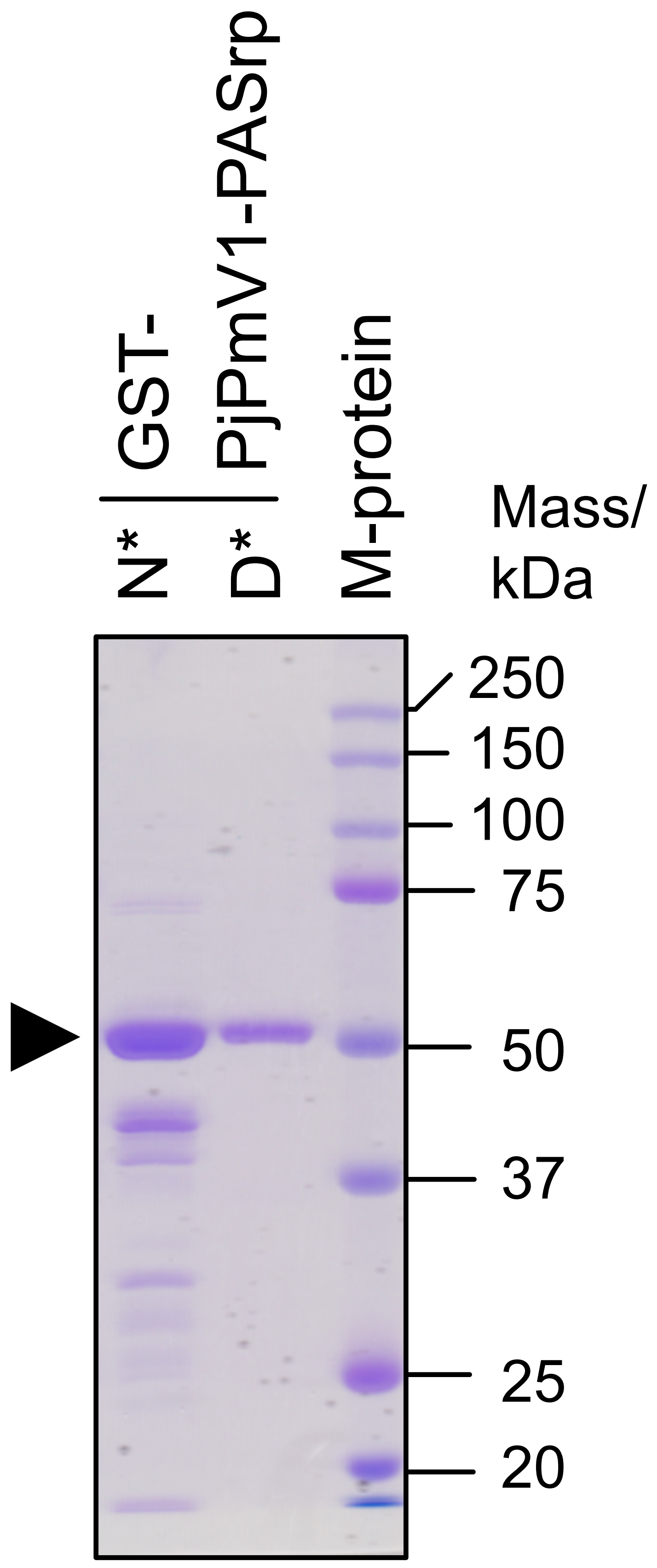
N*
D*
GST-
PjPmV1-PASrp
M-protein
Mass/
kDa
250
150
100
75
50
37
25
20

FIG S2

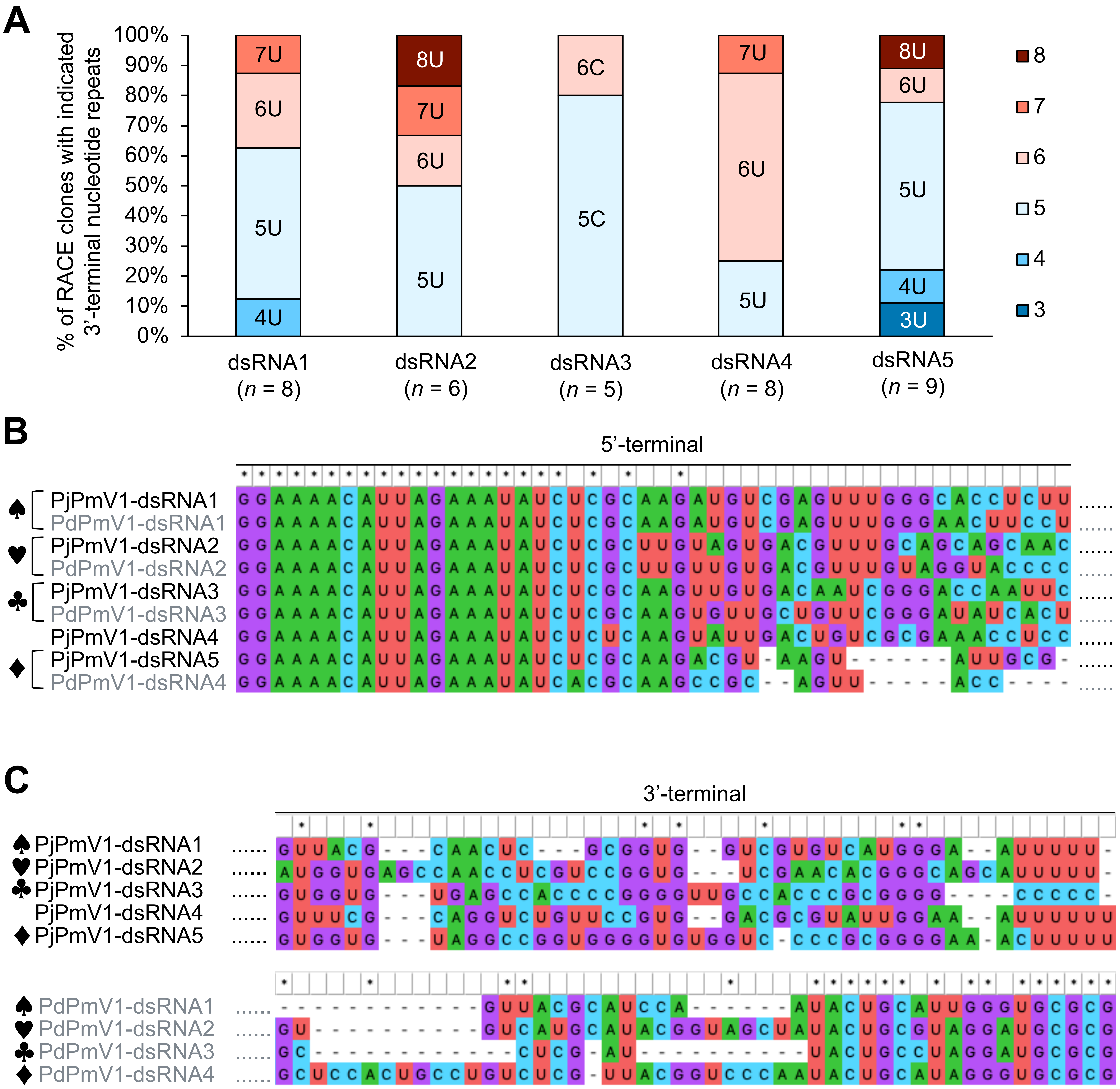

A
% of RACE clones with indicated 3'-terminal nucleotide repeats
100%
90%
80%
70%
60%
50%
40%
30%
20%
10%
0%
7U
6U
5U
4U
8U
7U
6U
5U
6C
5C
7U
6U
5U
8U
6U
5U
4U
3U
8
7
6
5
4
3
dsRNA1 (n = 8)
dsRNA2 (n = 6)
dsRNA3 (n = 5)
dsRNA4 (n = 8)
dsRNA5 (n = 9)
B
5'-terminal
PjPmV1-dsRNA1
PdPmV1-dsRNA1
PjPmV1-dsRNA2
PdPmV1-dsRNA2
PjPmV1-dsRNA3
PdPmV1-dsRNA3
PjPmV1-dsRNA4
PjPmV1-dsRNA5
PdPmV1-dsRNA4
C
3'-terminal
PjPmV1-dsRNA1
PjPmV1-dsRNA2
PjPmV1-dsRNA3
PjPmV1-dsRNA4
PjPmV1-dsRNA5
PdPmV1-dsRNA1
PdPmV1-dsRNA2
PdPmV1-dsRNA3
PdPmV1-dsRNA4

FIG S3

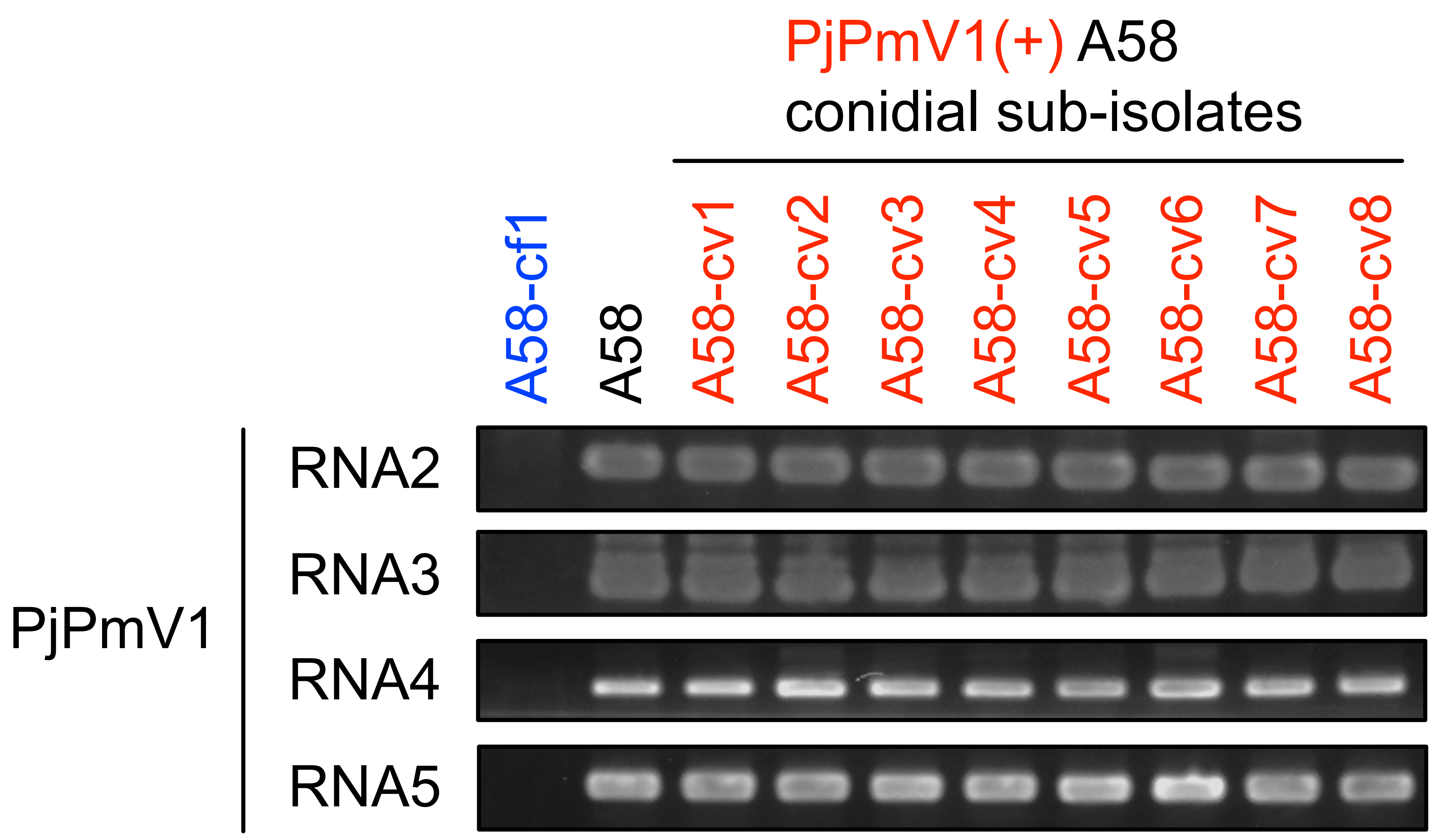

PjPmV1(+) A58
conidial sub-isolates
A58-cf1
A58
A58-cv1
A58-cv2
A58-cv3
A58-cv4
A58-cv5
A58-cv6
A58-cv7
A58-cv8
PjPmV1
RNA2
RNA3
RNA4
RNA5

FIG S4

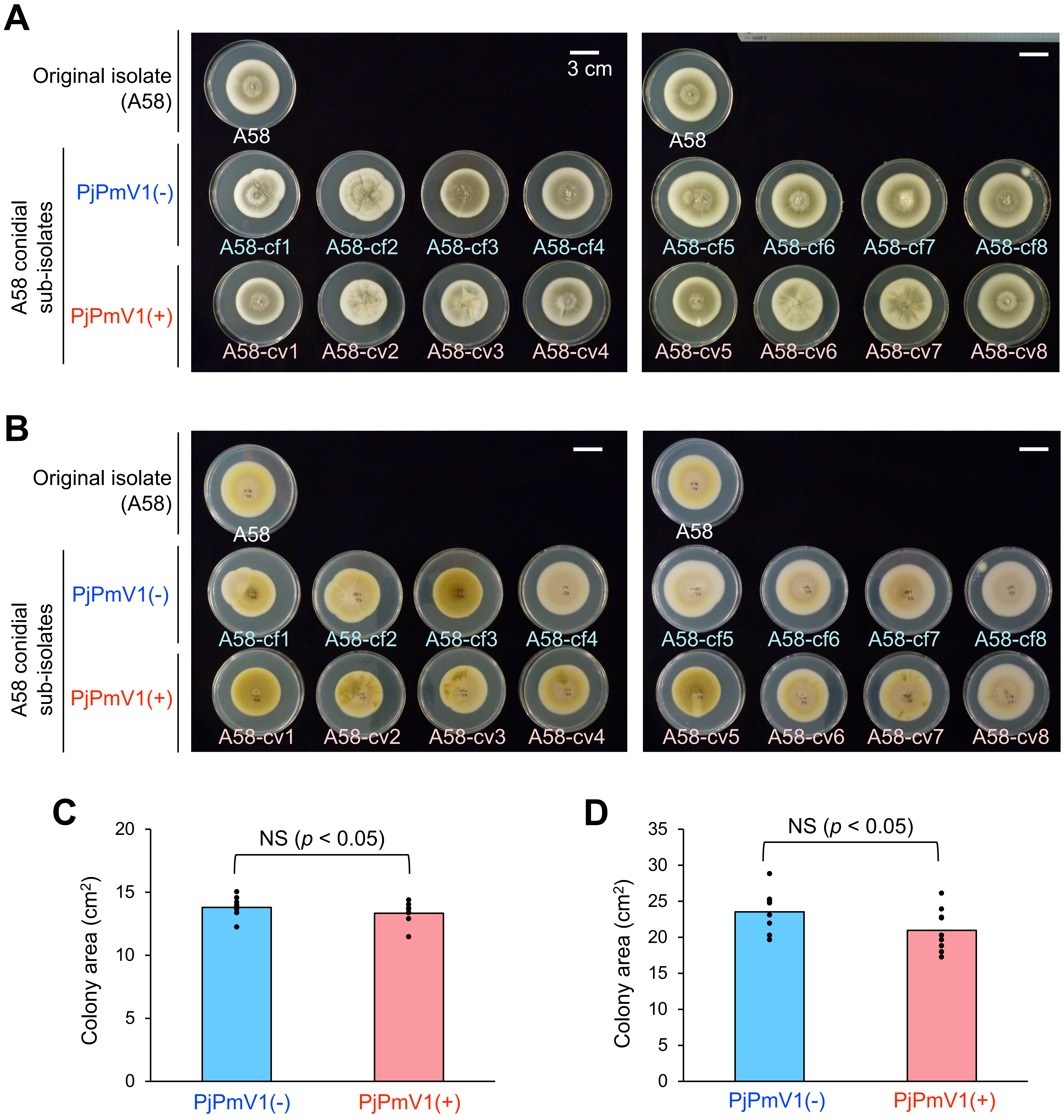
A
Original isolate
(A58)
A58 conidial
sub-isolates
PjPmV1(-)
PjPmV1(+)
3 cm
A58
A58-cf1
A58-cf2
A58-cf3
A58-cf4
A58-cf5
A58-cf6
A58-cf7
A58-cf8
A58-cv1
A58-cv2
A58-cv3
A58-cv4
A58-cv5
A58-cv6
A58-cv7
A58-cv8
B
Original isolate
(A58)
A58 conidial
sub-isolates
PjPmV1(-)
PjPmV1(+)
A58
A58-cf1
A58-cf2
A58-cf3
A58-cf4
A58-cf5
A58-cf6
A58-cf7
A58-cf8
A58-cv1
A58-cv2
A58-cv3
A58-cv4
A58-cv5
A58-cv6
A58-cv7
A58-cv8
C
Colony area (cm^2)
0
5
10
15
20
NS (p < 0.05)
PjPmV1(-)
PjPmV1(+)
D
Colony area (cm^2)
0
5
10
15
20
25
30
35
NS (p < 0.05)
PjPmV1(-)
PjPmV1(+)

FIG S5

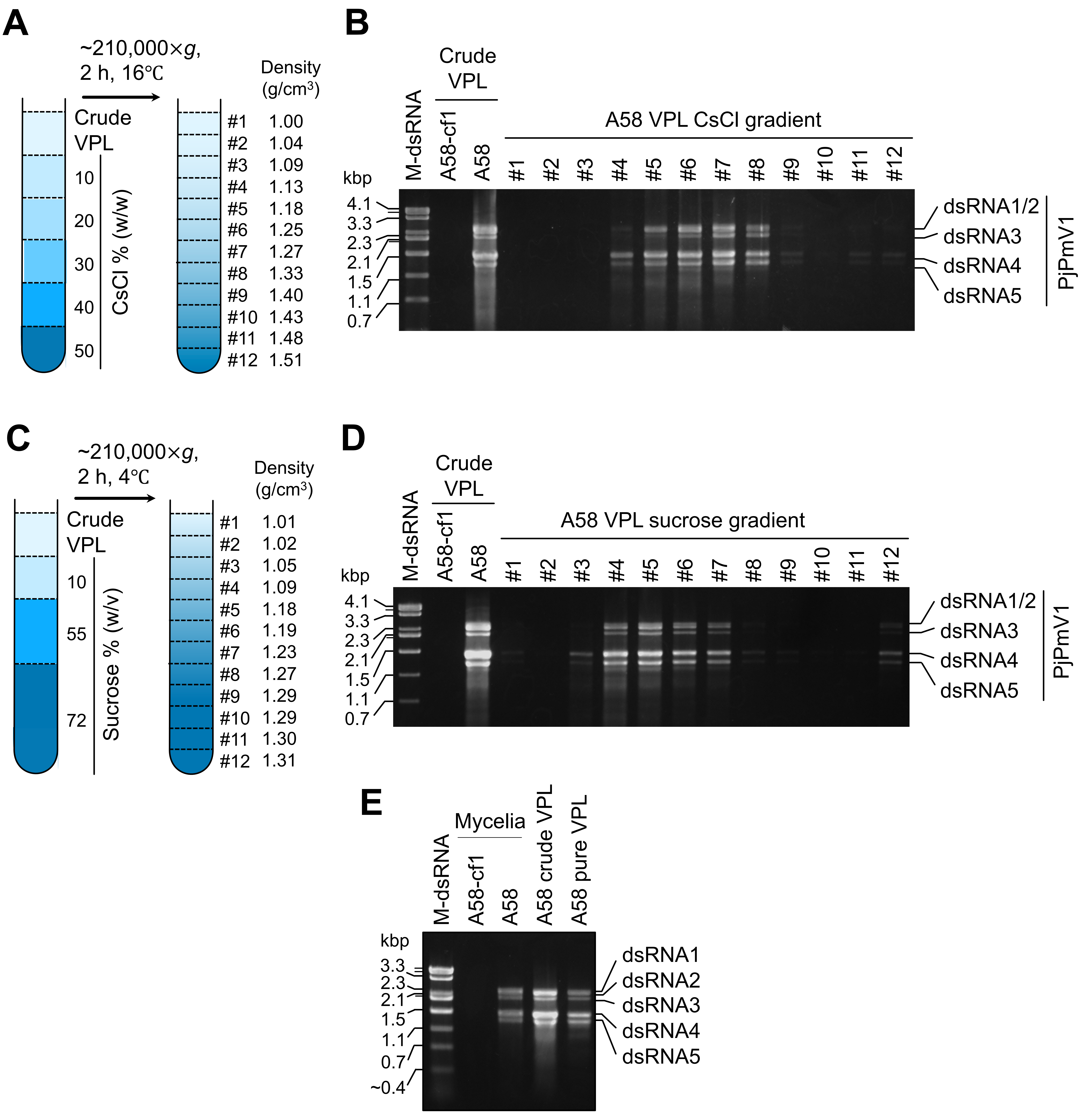
A
~210,000×g,
2 h, 16℃
Crude
VPL
10
20
30
40
50
CsCl % (w/w)
Density
(g/cm3)
#1 1.00
#2 1.04
#3 1.09
#4 1.13
#5 1.18
#6 1.25
#7 1.27
#8 1.33
#9 1.40
#10 1.43
#11 1.48
#12 1.51
B
Crude
VPL
M-dsRNA
A58-cf1
A58
A58 VPL CsCl gradient
#1
#2
#3
#4
#5
#6
#7
#8
#9
#10
#11
#12
kbp
4.1
3.3
2.3
2.1
1.5
1.1
0.7
dsRNA1/2
dsRNA3
dsRNA4
dsRNA5
PjPmV1
C
~210,000×g,
2 h, 4℃
Crude
VPL
10
55
72
Sucrose % (w/v)
Density
(g/cm3)
#1 1.01
#2 1.02
#3 1.05
#4 1.09
#5 1.18
#6 1.19
#7 1.23
#8 1.27
#9 1.29
#10 1.29
#11 1.30
#12 1.31
D
Crude
VPL
M-dsRNA
A58-cf1
A58
A58 VPL sucrose gradient
#1
#2
#3
#4
#5
#6
#7
#8
#9
#10
#11
#12
kbp
4.1
3.3
2.3
2.1
1.5
1.1
0.7
dsRNA1/2
dsRNA3
dsRNA4
dsRNA5
PjPmV1
E
Mycelia
M-dsRNA
A58-cf1
A58
A58 crude VPL
A58 pure VPL
kbp
3.3
2.3
2.1
1.5
1.1
0.7
~0.4
dsRNA1
dsRNA2
dsRNA3
dsRNA4
dsRNA5
